# Therapeutic Effects of Liposome-Enveloped *Ligusticum chuanxiong* Essential Oil on Hypertrophic Scars in the Rabbit Ear Model

**DOI:** 10.1371/journal.pone.0031157

**Published:** 2012-02-21

**Authors:** Hong Zhang, Xia Ran, Chang-Ling Hu, Lu-Ping Qin, Ying Lu, Cheng Peng

**Affiliations:** 1 Department of Pharmacognosy, School of Pharmacy, Second Military Medical University, Shanghai, People's Republic of China; 2 Key Laboratory of Standardization of Chinese Herbal Medicines of Ministry of Education, Pharmacy College, Chengdu University of Traditional Chinese Medicine, Chengdu, People's Republic of China; 3 School of Life Science, East China Normal University, Shanghai, People's Republic of China; 4 Department of Pharmaceutics, School of Pharmacy, Second Military Medical University, Shanghai, People's Republic of China; Duke University Medical Center, United States of America

## Abstract

Hypertrophic scarring, a common proliferative disorder of dermal fibroblasts, results from an overproduction of fibroblasts and excessive deposition of collagen. Although treatment with surgical excision or steroid hormones can modify the symptoms, numerous treatment-related complications have been described. In view of this, we investigated the therapeutic effects of essential oil (EO) from rhizomes of *Ligusticum chuanxiong* Hort. (Umbelliferae) on formed hypertrophic scars in a rabbit ear model. EO was prepared as a liposomal formulation (liposome-enveloped essential oil, LEO) and a rabbit ear model with hypertrophic scars was established. LEO (2.5, 5, and 10%) was applied once daily to the scars for 28 days. On postoperative day 56, the scar tissue was excised for masson's trichrome staining, detection of fibroblast apoptosis, assays of the levels of collagens I and III, and analysis of the mRNA expression of matrix metalloproteinase-1 (MMP-1), caspase-3 and -9, and transforming growth factor beta 1 (TGF-β_1_). In addition, the scar elevation index (SEI) was also determined. As a result, LEO treatment significantly alleviated formed hypertrophic scars on rabbit ears. The levels of TGF-β_1_, MMP-1, collagen I, and collagen III were evidently decreased, and caspase -3 and -9 levels and apoptosis cells were markedly increased in the scar tissue. SEI was also significantly reduced. Histological findings exhibited significant amelioration of the collagen tissue. These results suggest that LEO possesses the favorable therapeutic effects on formed hypertrophic scars in the rabbit ear model and may be an effective cure for human hypertrophic scars.

## Introduction

Hypertrophic scarring generally occurs following surgery, trauma and especially burns, which is a common proliferative disorder of dermal fibroblasts and results from an overproduction of collagen and excessive deposition of extracellular matrix [1]. Patients with hypertrophic scars often report itching and pain, and experience serious functional and cosmetic problems, which are caused by a variety of complications, including compression, sensation of stiffness, loss of joint mobility and anatomical deformities [2], [3].

When a deep wound or an exuberant scar occurs, there are many preventive and therapeutic measures such as silicone, pressure therapy, corticosteroids, laser therapy, cryotherapy, radiation, surgery, and some new methods developed such as interferon and 5-fluorouracil. However, there is no consensus about the best treatment to produce complete and permanent improvement of scars with few side effects [4], [5].

Natural drugs have been generally recognized to play a unique therapeutic role in the treatment of many diseases [6]–[9]. Essential oils from many plant species have become especially popular in recent years, and investigations into their bioactivities and mechanisms of action have been performed for human health [10], [11].

Rhizoma Chuanxiong, the rhizome of *Ligusticum chuanxiong* Hort. (Umbelliferae) and one of the well-known traditional Chinese medicines, is warm in property and pungent in flavor and possesses efficacy in promoting the circulation of the blood and qi, expelling wind, and alleviating pain, which has high medicinal value. It has been used in the clinical treatment of pain, migraine, rheumatic arthralgia, inflammation, menstrual disturbance, and cardiovascular and cerebrovascular diseases for nearly 2000 years [12]–[14]. The volatile compounds in this herbal drug are considered an important part of its pharmacological effects mentioned above. There are several investigations reported suggesting that the essential oil (EO) from this plant could protect against DNA damage and apoptosis induced by ultraviolet B in mammalian cells [15]. The Ethyl ether extract blocks endothelial cell damage induced by hydrogen peroxide [16]. The anti-Trichophyton effects of *Ligusticum chuanxiong* essential oil are also validated [17].

Our previous preliminary investigations indicated that EO extracted from the plant markedly suppressed the viability of human hypertrophic scar fibroblasts (HSFs), elicited cell apoptosis *in vitro* [18], and inhibited hypertrophic scar formation in the rabbit ear model [19], suggesting its preventive effects on hypertrophic scarring. Nevertheless the good preventive effect of a drug does not mean its favorable therapeutic action. As far as we are aware no investigators have reported the therapeutic effects of EO on produced hypertrophic scars. This study was designed to demonstrate whether EO could alleviate or eliminate formed hypertrophic scars in the rabbit ear model and to explore the possible mechanism of action.

## Results

### LEO inhibits the formation of collagen I and collagen III

**Figure 1:**
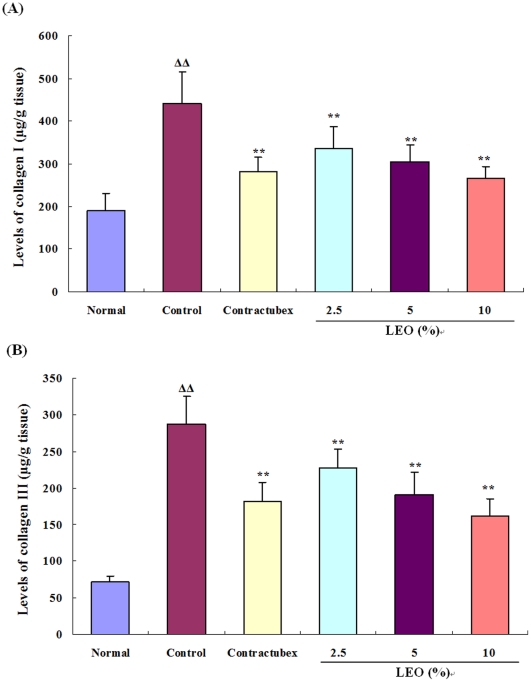
Effects of liposome-enveloped essential oil (LEO) on the levels of collagens I (A) and III (B). The levels of collagens I and III in the control group significantly increased after 56 postoperative days when compared with the normal group. The treatment with LEO for 28 days decreased the levels of collagens I and III dramatically and dose-dependently in comparison with the control group. ΔΔ P<0.01 compared with the normal group; **P<0.01 compared with the control group. Data are expressed as the mean ± SD. n = 12.

Collagens, including collagen I and collagen III, are the most important ingredients of ECM. As shown in Figure 1A and 1B, the levels of collagens I and III were significantly increased in the control group when compared with the normal group. By contrast, in the LEO treatment groups, the levels of collagens I and III decreased dramatically after 28 days of the treatment with LEO, and in the highest group, the levels of collagens I and III were even lower than those in the contractubex group.

### mRNA levels of MMP1, TGF-β_1_, caspase-3 and caspase-9

**Figure 2:**
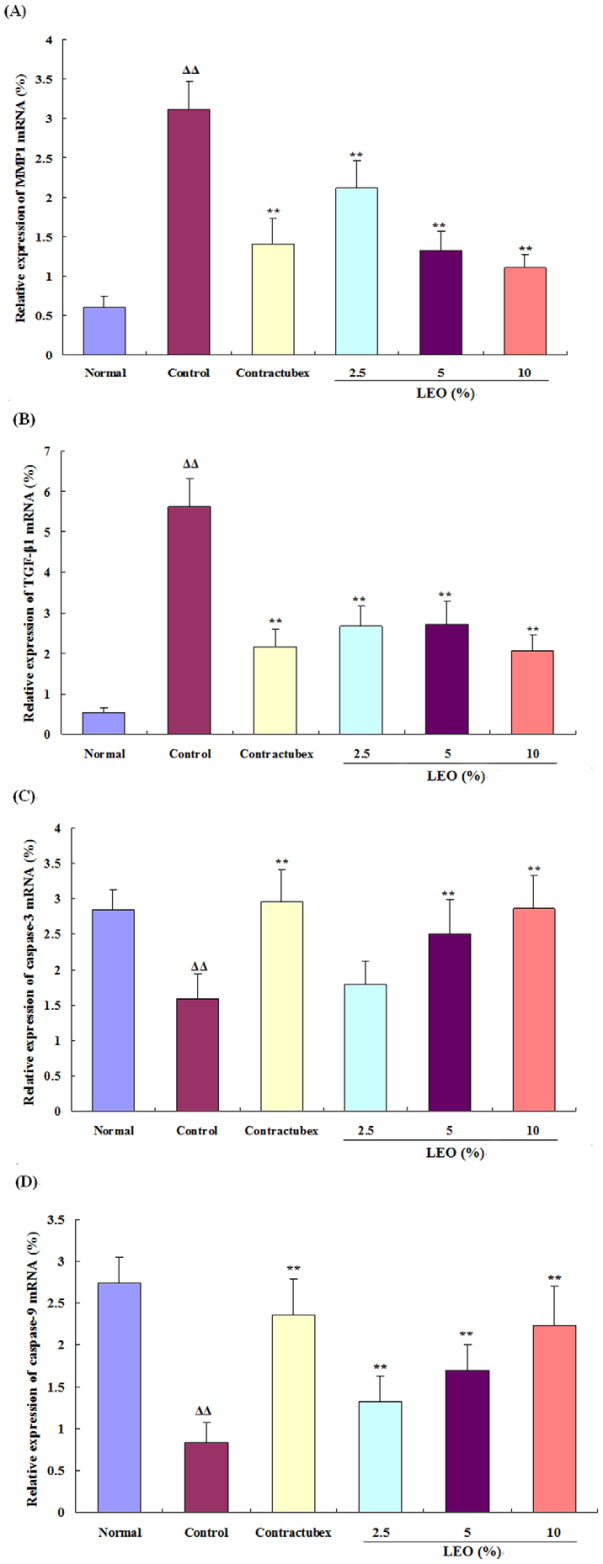
The changes of relative mRNA expression levels of MMP1, TGF-β_1_, caspase-3, and caspase-9. The mRNA expression levels of MMP1 and TGF-β_1_ significantly increased in the control group after 56 postoperative days when compared with the normal group, while evidently decreased in the groups treated with LEO for 28 days in comparison with the control group. On the other hand, the mRNA expression levels of caspase-3 and caspase-9 in the control group decreased dramatically after 56 postoperative days when compared with the normal group. The treatment with LEO for 28 days significantly increased the mRNA expression levels of caspase-3 and caspase-9 when compared with the control group. ΔΔP<0.01 compared with the normal group; **P<0.01 compared with the control group. Data are expressed as the mean ± SD. n = 12.

MMP1 and TGF-β_1_ play important roles in the formation of scars. Their mRNA expression levels significantly ascended in the control group in comparison with the normal group, but markedly decreased in the groups treated with the doses of 2.5, 5, and 10% LEO for 28 days (Figue 2A and 2B). Activation of caspase-3 and caspase-9 can evidently induce apoptosis of the hypertrophic fibroblasts. In this study, their mRNA expression levels were reduced in the control group when compared with the normal group, while increased dramatically and dose-dependently in the treatment groups given the doses of 2.5, 5, and 10% LEO for 28 days (Figure 2C and 2D).

### Quantification of TUNEL positive cells

**Figure 3:**
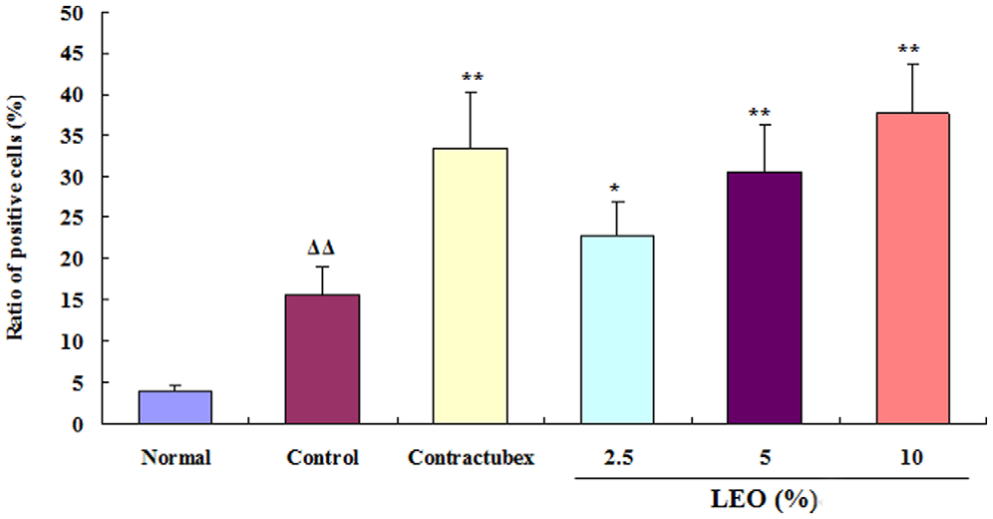
Effects of LEO on the ratio of TUNEL positive scar fibroblasts. After 56 postoperative days, the amount of TUNEL positive scar fibroblasts in scar tissue markedly increased in the control group in comparison with the normal group. Nevertheless the treatment with LEO for 28 days evidently increased the number of TUNEL positive scar fibroblasts when compared with the control group. ΔΔP<0.01 compared with the normal group; *P<0.05, **P<0.01 compared with the control group. Data are expressed as the mean ± SD. n = 12.

Sections from rabbit ears subjected to liposomes with or without EO or contractubex were stained for visualization of fragmented DNA, which indicated apoptosis cells. One of the characteristics of hypertrophic scarring is abnormal proliferation of dermal fibroblasts, but LEO significantly induced apoptosis of scar fibroblasts in this study. As shown in Figure 3, the ratio of TUNEL positive cells in the control group was higher than that in the normal group, indicating an increase in apoptosis of scar fibroblasts, but significantly lower than that in the groups treated with the doses of 2.5, 5, and 10% LEO for 28 days, suggesting that LEO elicited more apoptosis of scar fibroblasts.

### Scar elevation index

**Figure 4:**
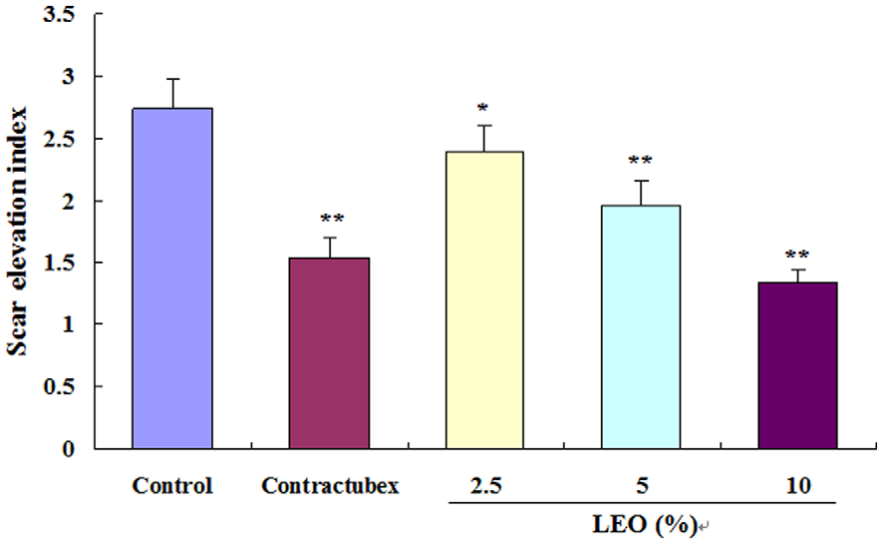
LEO decreases scar elevation index. The degree of scar hypertrophy is reflected by scar elevation index (SEI), which represents the ratio of total scar connective tissue area to the area of underlying dermis. After treatment with LEO for 28 days, the formed hypertrophic scars decreased in a dose-dependent manner. *P<0.05, **P<0.01 versus the control group. Data are expressed as the mean ± SD. *n* = 12.

On day 56 post-wounding, there was significant hypertrophic scarring in the control group with the mean scar elevation index (SEI) of 2.74±0.24 (Figure 4). Successive administration of LEO for 28 days since day 29 post-wounding markedly and dose-dependently inhibited scar hyperplasia in the treatment groups with the mean SEI of 2.39±0.21, 1.96±0.20, and 1.34±0.11, respectively. SEI in the highest group (10% LEO) was even lower than that in the contractubex group (1.53±0.17).

### Masson's trichrome staining findings

**Figure 5:**
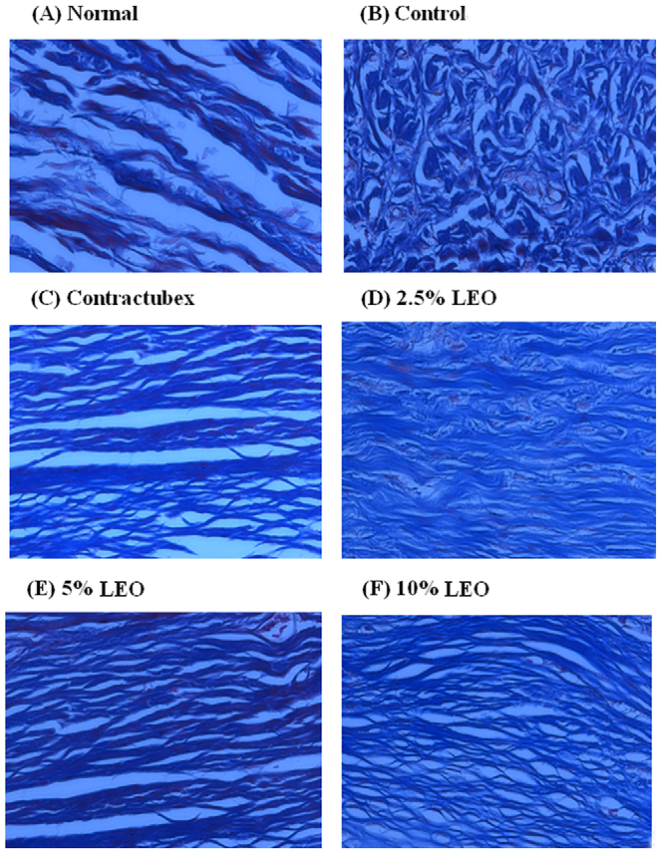
Masson's trichrome staining findings for collagen. (A) Unwounded (normal) skin with regularly arranged, small collagen fibers. (B) In the control group treated with empty liposomes, the amount of collage fibers is far more than that in the normal group, and collage fibers are thicker, denser and disorganized. (C) Contractubex. (D–F) After treatment with (D) 2.5%, (E) 5% and (F) 10% LEO for 28 days, the collagen fibers were arranged more regularly and sparse than those in the control group. Original magnification ×400.

Overproduction of collage is another characteristic of hypertrophic scars. Masson staining of scar tissue was carried out on day 56 post-wounding. Light microscopic examination revealed typical features of collagen fibers in scar tissue (Figure 5B) in the control group compared with unwounded dermal tissue (Figure 5A). The collagen bundles were thicker, denser, disorganized and more abundant. By contrast, collage fibers were decreased, thinner and more regularly ranged in the groups treated with LEO, which found dose dependent (Figure 5D–F).

## Discussion

Generally researchers are apt to investigate the preventive effects of natural drugs on hypertrophic scar formation, but it dose not reflect their therapeutic actions authentically. After all, drugs are more commonly used to treat formed hypertrophic scars clinically and the good preventive effect of a drug does not mean its favorable therapeutic action. Therefore we further investigated the therapeutic effect of the essetial oil (EO) from *Ligusticum chuanxiong* on formed hypertrophic scars in the rabbit ear model on the basis of our previous preliminary investigations [18], [19] for a show of its clinical value.

Liposomes are artificially prepared vesicles made of lipid bilayer, which can be filled with drugs, and have been extensively employed to enhance the efficiency of drug delivery via several routes of administration. liposomes can facilitate deposition of drugs into the skin and significantly enhance the accumulation of drugs at the site of administration as a result of the high substantivity of liposomes with biological membranes. liposomal drug formations have been substantiated to be markedly superior to conventional dosage forms, especially for the topical mode of administration of drugs [20]. Thus the essential oil (EO) was prepared as a liposomal formulation (LEO) for use in this study.

The rabbit ear model with hypertrophic scars was first established by Morris et al. in 1997 [21]. After that this model was modified further by many researchers [22]–[24] for its transient hypertrophy. Currently this model provides reproducible cutaneous hypertropic scars which are in parallel with human scar on the whole in gross appearance and histological features lasting over 3 months, and are commonly used for the investigation of anti-scarring modalities, such as steroid treatment, silicone occlusion, and procollagen C-proteinase inhibitor therapy. Our previous study showed significant scarring on the rabbit ears from postoperative day 28 to day 56, which is similar to previous reports [22]–[24], so the drug treatment was carried out duing this period in the model rabbits in the present study.

Hypertrophic scarring is characterized by overproduction of fibroblasts and excessive deposition of collagen. Hypertrophic scar fibroblasts (HSFs) have a higher capacity for proliferation, cytokine production, and collagen synthesis than normal fibroblasts [25]. Therefore, inhibitory proliferation or induction of apoptosis in fibroblasts is an important treatment for hypertrophic scarring. Our previous study indicated that EO markedly induced apoptosis of HSFs from human skin tissue [19]. In the present study, in situ detection of cell apoptosis was performed to further substantiate whether LEO facilitated HSFs apoptosis in scar tissue. It is interesting that the ratio of TUNEL positive cells had a significant increase in LEO treatment groups compared with the control group, suggesting that LEO induced more apoptosis of scar fibroblasts.

Collagen, secreted mainly by fibroblasts, is the main component of extracellular matrix (ECM) and the principal structural protein holding the skin. Collagen dysmetabolism is the pathological basis of hypertrophic scarring [26], [27] and is also a common cause of fibrotic diseases. Normal fibroblasts synthesize both collagen I and collagen III, but this synthesis becomes imbalanced with changes in the surrounding environment. The immature and instable type III collagen is typically seen during the early phase of wound healing, while the predominant collagen type in skin is the mature and mechanically stable type I collagen [28]. During granulation tissue formation, type III collagen expression increases more than the type I expression [29], but much of type III collagen is replaced later by the stronger and tougher type I collagen. Our results also demonstrated that the amount of type I collagen was more than that of type III collagen in the formed hypertrophic scar tissue of the model rabbit ears on postoperative day 56. LEO dramatically and dose-dependently suppressed types I and III collagen protein synthesis in the hypertrophic scar tissue of the rabbit ears. Pathological examination also showed that collagen fibers evidently decreased in LEO treatment groups compared with the control group.

We further observed the mRNA expression levels of matrix metalloproteinase-1 (MMP-1), transforming growth factor beta 1 (TGF-β_1_) in scar tissue, which are closely related to overproduction of fibroblasts and excessive deposition of collagen. Wound repair involves cell migration, proliferation, and tissue remodeling. These ordered and regulated processes are facilitated by matrix-degrading proteases. Collagenase is the only known enzyme able to start the breakdown of the interstitial collagens (types I, II and III). MMP-1 plays a key role in the remodelling that occurs constantly in both unwounded and diseased conditions [30], and is the key enzyme in the degradation of type I and type III collagens in scars. There is considerable evidence that a number of cytokines are important components in the process of wound healing and scar formation. One of the most intensively investigated molecules associated with many types of fibrosis is TGF-β_1_, which stimulates infiltration of inflammatory cells and fibroblasts, and induces fibroblast proliferation, angiogenesis and synthesis of ECM, while a persistent autocrine loop of TGF-β_1_ contributes to hypertrophic scar formation [31]. In this study, the expression of MMP-1 and TGF-β_1_ was significantly increased in the control group of scar tissue. It seems inconsistent in terms of their effects. Presumably they maintained a dynamic balance at higher levels. Excessive TGF-β_1_ facilitated overproduction of scar fibroblasts, while scar fibroblasts further secreted more MMP-1 to degrade collagen protein. LEO evidently decreased the expression levels of MMP-1 and TGF-β_1_ mainly by induction of apoptosis in scar fibroblasts.

Apoptotic cell death is mediated by molecular pathways that culminate in the activation of a family of cysteine proteases, known as the caspases, which orchestrate the dismantling and clearance of dying cells [32], [33]. There is considerable evidence demonstrating that activation of caspase-9 and -3 has significant relationships with scar fibroblast apoptosis. Caspase-3 activity has been described to be essential for drug-induced apoptosis, and caspase-9 is necessary for the process of apoptosis in scar fibroblasts [34], [35]. In the process of cell apoptosis, pro-caspase-9 is recruited to the “apoptosome” complex, while the recruitment causes caspase-9 cleavage and further activates downstream executioner caspases such as caspase-3 [36]–[38]. The activation of caspase-9 and -3 via the release of cytochrome c can induce apoptosis in scar fibroblasts in vivo [35], [39]. In order to further verify apoptosis in scar fibroblasts, we detected the mRNA expression levels of caspase-3 and caspase-9. After treatment with LEO for 28 days, the mRNA expression of caspase-3 and -9 increased markedly and dose-dependently compared with the control group, suggesting that LEO facilitated fibroblast apoptosis in scar tissue.

In a previous study, we also found that EO significantly inhibited the growth of cultured HSFs, and induced cell apoptosis [18]. Based on these findings, we deduce that LEO alleviates formed hypertrophic scars mainly through inhibition of HSF proliferation and induction of HSF apoptosis, as the abnormal biological behaviour of fibroblasts plays a central role in hypertrophic scar formation and development [40]. Fibroblasts synthesize both type I and type III collagens, which are the main components of the ECM, and also excrete TGF-β_1_ and MMP-1, which regulate the synthesis and degradation of collagen.

In conclusion, LEO alleviates produced hypertrophic scars in the rabbit ear model, mainly by inhibiting HSF proliferation and inducing HSF apoptosis, which further downregulates the mRNA expression of TGF-β_1_ and MMP-1, and upregulates degradation of collagen types I and III. LEO is potentially an effective cure for human hypertrophic scarring.

## Materials and Methods

### Ethics statement

All animal treatments were strictly in accordance with international ethical guidelines and the National Institutes of Health Guide concerning the Care and Use of Laboratory Animals, and the experiments were carried out with the approval of the Animal Experimentation Ethics Committee of the Second Military Medical University.

### Drug preparation

In accordance with our previous reports [18], [19], the essential oil (EO) was extracted and its chemical composition was analyzed by GC-MS. Dozens of compounds were detected from EO and the major components were ligustilide and butylidenephthalide with relative contents of 67.027 and 5.831%, respectively. In this study, a liposome formulation was used due to its favorable penetrability and topical accumulation. Liposome-eneloped EO (LEO) was prepared by Dr. Ying Lu, an associate Professor from Department of Pharmaceutics, School of Pharmacy, Second Military Medical University (Shanghai, China), and empty liposomes were used as placebo.

### Rabbit ear model of hypertrophic scar tissue

A previously reported hypertrophic scar rabbit model was used in this study [41]. Six female New Zealand white rabbits, (Shanghai Si-Lai-Ke Experimental Animal Co. Ltd., Shanghai, China), with an initial body weight of 2.0±0.2 kg, were used. Animals were housed in a regulated environment (22±2°C), with a 12 h light/dark cycle (light cycle was 08.00–20.00 h). Food and water were given *ad libitum* throughout the experiment.

The animals were intravenously anaesthetized with sodium pentobarbital 30 mg/kg. Under sterile conditions, six full-thickness wounds were created down to the bare cartilage on each ear using a biopsy punch. Each ear received six round wounds, 10 mm in diameter, placed in a random order on the ear, thus each rabbit had 12 wounds. The epidermis, dermis and perichondrium in each wound were thoroughly removed, and the wounds were covered with sterile gauze for 1 day. After recovery from anaesthesia, the rabbits were returned to their cages.

### Grouping and treatment

On postoperative day 29 and afterwards, scars were randomly divided into five groups, with 12 scars to each group: one control (placebo) group, three LEO treatment groups and one positive group treated with contractubex, known to reduce exuberant collagen production (Merz Pharma GmbH, Frankfurt, Germany). The scars in the control group were thinly coated with liposomes without EO once a day. LEO (2.5%, 5%, and 10%, w/v) was applied once daily to the scars in the three treatment groups, and contractubex to the scars in the positive group. One further unwounded rabbit with full-thickness skin on its ears (Normal group) was used as a further (unwounded) placebo, and received neither liposomes (with or without EO) or contractubex.

### Determination of collagen I and collagen III

All animals were killed on posttherapeutic day 28 (postoperative day 56), the scar tissue was separated from the rest of the tissue, and the cartilage removed. The weighed scar tissue was diced, and rapidly frozen in liquid nitrogen until used. Once the tissue was removed from the liquid nitrogen, it was maintained at 4°C after thawing. To each 50 mg of tissue, 1 mL of phosphate-buffered saline (pH 7.4) was added, then the tissue was homogenized and separated by centrifugation at 3000 *g* and 4°C for 20 min, and the supernatant was collected for assay.

Collagen I and collagen III were measured by ELISA using an appropriate commercial ELISA kit for each, according to the manufacturer's instructions (R & D Systems Inc., Minneapolis, MN, USA). Briefly, 40 µL of sample dilution and 10 µL of the supernatant were added to a testing sample well, gently mixed, and incubated for 30 min at 37°C after closing the microplate with the membrane supplied with the kit, after which the membrane was removed and the liquid in the well discarded. Washing buffer was added to the well and left for 30 s, then drained. This procedure was repeated five times, then 50 µL of horseradish peroxidase-conjugated reagent was added to each well and incubated for 30 min at 37°C, followed by washing five times with washing buffer from the kit. Each well was incubated with chromogen solution A (50 µL) and chromogen solution B (50 µL) for 15 min at 37°C, and the stop solution (50 µL) added to each well to terminate the reaction. The optical density was measured at 450 nm using a microplate reader (ELx-800 Universal; Bio-Tek, North Brunswick, NJ, USA).

### RNA isolation and Fluorescent quantitative reverse transcription-PCR (FQ-RT-PCR)

TriPure Isolation Reagent (Roche Diagnostics, Vilvoorde, Belgium) was used to extract the total mRNA of the scar tissue in accordance with our previous report (17), and the isolated RNA was treated with RNase-free DNase (Promega). Reverse transcription was performed using a cDNA synthesis kit according to the manufacturer's instructions (Applied Biosystem).

Primer pairs for rabbit genes (MMP1, TGF-β_1_, Caspase-3, Caspase-9) were designed using the Primer Express design software (Applied Biosystems) and listed in Table 1. The house-keeping gene GAPDH was used as an internal control. FQ-RT-PCR was performed on a real-time PCR instrument (ABI 7900HT, Applied Biosystems) for 40 cycles consisting of denaturation at 95°C for 30 s, annealing at 59°C for 30 s and extension at 72°C for 30 s. All amplifications and detections were carried out in a MicroAmp optical 384-well reaction plate with optical adhesive covers (Applied Biosystems). Relative expression of mRNA (%) = 2^−ΔCT(1,2,3,4)^×100%, where CT represents threshold cycle, ΔCT_1_ = CT_(MMP1)_−CT_(GAPDH)_, ΔCT_2_ = CT_(TGF-β1)_−CT_(GAPDH)_, ΔCT_3_ = CT_(Caspase-3)_−CT_(GAPDH)_, ΔCT_4_ = CT_(Caspase-9)_−CT_(GAPDH)_

**Table 1:** Primers used in real-time RT-PCR analysis.

Gene	Primer sequence	Species	Amplicon size (bp)
MMP1	S:5′ttccaaagcagagaggcaat 3′	Rabbit	84
	A:5′gcttcccagtcactttcagc 3′		
TGF-β_1_	S: 5′ acattgacttccgcaaggac 3′	Rabbit	104
	A: 5′ gtccaggctccagatgtagg 3′		
Caspase-3	S: 5′ tgccatgctgaaagagtacg 3′	Rabbit	104
	A: 5′ tggcatcaagggaataggac 3′		
Caspase-9	S: 5′ ccaggtggatgctgtgtcta 3′	Rabbit	170
	A: 5′ gcagggactgtaggtcttcg 3′		
GAPDH	S: 5′ caacgaatttggctacagca 3′	Rabbit	183
	A: 5′ ctgtgaagaggggcagattc 3′		

### Detection of apoptotic cells by TUNEL assay

For in situ detection of DNA fragmentation in paraffin-embedded tissue sections, the TUNEL method was performed using the TUNEL Apoptosis Detection Kit (KeyGen Biotech Co., Nanjing, China), following manufacturer's instructions. Briefly, the tissue sections were deparaffinized with dimethylbenzene, rehydrated through gradient ethanol immersion, and then incubated in working solution comprising 2 µL 50×proteinase K and 98 µL PBS for 20 min at room temperature for Protein digestion. Endogenous peroxidase was inactivated with 3% H_2_O_2_ in methanol for 10 min at room temperature. The reaction fluid containing dUTP and TdT enzyme was added to the sections and incubated at 37°C in a humified chamber for 1 h. The sections were rinsed with PBS 3 times, appended 50 µL streptavidin-HRP treatment fluid, and incubated for 30 min at 37°C in a humified chamber. Following rinsing with PBS 3 times, the sections were incubated in working solution containing diaminobenzidine and 3% H_2_O_2_ for 10 min. Finally, the sections were washed with PBS, counterstained in haematoxylin, dehydrated and observed in a light microscope. The positive cells were counted and evaluated as the ratio of TUNEL-positive cells to a total number of fibroblast cells.

### Determination of SEI

Scar tissue with cartilage was fixed with 10% buffered formalin for 3 days, embedded in paraffin, sectioned with a dermatome, and stained using hematoxylin–eosin (H&E). Light microscopy was used to examine the degree of scar hyperplasia, which was expressed as the scar elevation index (SEI). SEI represents the ratio of the scar tissue height to the normal tissue below the hypertrophic scar. A ratio of 1 indicates no difference in the wound area height compared with unwounded skin.

### Masson's trichrome staining for collagen fibers

Scar tissue was fixed with 10% formalin, embedded in paraffin, sectioned and stained with Masson's Trichrome Stain Kit following the manufacturer's instructions (KeyGen Biotech Co., Nanjing, China). Briefly, the tissue sections were deparaffinized with dimethylbenzene, rehydrated through gradient ethanol immersion, and washed in running water for 5 minutes. The tissue was stained in Weigert's iron hematoxylin working solution for 10 minutes before rinsing the sections for 5 minutes in running water and washing them in distilled water. The slides were stained in Biebrich scarlet-acid fuchsin solution for 5 minutes, washed in distilled water, and placed in 1% phosphomolybdic-phosphotungstic acid solution for 5 minutes. Subsequently, the sections were transferred directly into aniline blue solution, stained for 5 minutes, and rinsed in 1% acetic acid solution for 2 minutes. Finally, the tissue was dehydrated, cleared, and mounted. Collagen fibers were stained in blue.

### Statistical analysis

All results were presented as the mean ± SD. Data were analyzed using a SPSS 13.0 statistical package. Data for multiple comparisons were performed by one-way ANOVA followed by Dunnett's test. A value of P<0.05 was considered statistically significant.
